# The Number of X Chromosomes Influences Inflammatory Cytokine Production Following Toll-Like Receptor Stimulation

**DOI:** 10.3389/fimmu.2019.01052

**Published:** 2019-05-09

**Authors:** Nicolas Lefèvre, Francis Corazza, Joseph Valsamis, Anne Delbaere, Viviane De Maertelaer, Jean Duchateau, Georges Casimir

**Affiliations:** ^1^Department of Pulmonology, Allergology and Cystic Fibrosis, Hôpital Universitaire des Enfants Reine Fabiola, Université Libre de Bruxelles, Brussels, Belgium; ^2^Laboratory of Translational Research, Université Libre de Bruxelles, Brussels, Belgium; ^3^Laboratory of Hormonology, Hôpital Universitaire Brugmann, Université Libre de Bruxelles, Brussels, Belgium; ^4^Fertility Clinic, Hôpital Erasme, Université Libre de Bruxelles, Brussels, Belgium; ^5^Department of Biostatistics and Medical Computing, Université Libre de Bruxelles, Brussels, Belgium; ^6^Laboratory of Pediatrics, Université Libre de Bruxelles, Brussels, Belgium

**Keywords:** sex differences, cytokine, Toll-like receptors, X chromosome, sex steroids

## Abstract

Sex differences are observed in the evolution of numerous inflammatory conditions. Women exhibit better clinical courses compared to men in acute inflammatory processes, yet worse prognosis in several chronic inflammatory diseases. Inflammatory markers are significantly different between prepubertal boys and girls, whose sex steroid levels are very low, suggesting genetics play a role. To evaluate the potential influence of the X chromosome, we studied cytokine production and protein phosphorylation following Toll-like receptor (TLR) activation in whole blood and purified neutrophils and monocytes of healthy adults of both sexes as well as subjects with Klinefelter syndrome. We recorded higher levels of inflammatory cytokines in men compared to both women and patients with Klinefelter syndrome following whole blood stimulation. In purified monocytes, production of inflammatory cytokines was also higher in men compared to women, while Klinefelter subjects expressed the same pattern of cytokine production as males, in contrast with whole blood analyses. These differences remained after adjusting for sex steroid levels. Our study revealed higher cytokine inflammatory responses in men than women, yet also compared to subjects with Klinefelter syndrome, who carry two copies of the X chromosome, like women, and thus potentially benefit from the cellular mosaicism of X-linked genes.

## Introduction

Sex differences are observed in the evolution of numerous inflammatory conditions in several mammalian species, including humans. In acute inflammatory processes, such as sepsis or burns, women have a tendency to exhibit better clinical courses and increased survival compared to men (1–3). On the other hand, women exhibit worse prognosis in several chronic inflammatory conditions, such as severe asthma, chronic obstructive pulmonary disease (COPD), and cystic fibrosis and sickle cell disease(CF) (4–7).

These sex-dependent differences probably depend on several mechanisms involving both hormonal and genetic factors (8–12). Sex hormones modulate both humoral and cell-mediated immune pathways during inflammatory responses (13, 14). It is common knowledge that the clinical differences between men and women in terms of inflammatory markers can be explained by their levels of sex steroids (15–17). However, sex hormone levels are very low before puberty, and recent studies have shown significant differences in immune markers between prepubertal boys and girls suffering from acute or chronic inflammatory diseases (18, 19). These findings are supporting the hypothesis that sex steroid levels are not sufficient to explain the sex differences observed during inflammatory conditions between different ages, from neonates to the elderly, suggesting that another mechanism is at work (20).

Numerous proteins involved in immune processes are encoded on the X chromosome (21). The genes of the some of the main protein members of the nuclear factor-κB (NF- κB) signaling pathway are linked to the X chromosome, namely interleukin (IL)-1 receptor-associated kinase 1 (IRAK-1), NF-κB essential modulator (NEMO), and Bruton's tyrosine kinase (BTK) (22).

One of the two X chromosomes carried by females is randomly inactivated during early embryogenesis in order to maintain the same dosage of proteins across both sexes. Females are thus composed of a mosaic of cells, from either the paternal or maternal X chromosome, providing them with greater diversity of responses against attack than males (22–25). Besides this phenomenon of mosaicism, approximately 15% of X-linked genes escape this process of methylation, thus increasing the X-linked cellular protein content in females compared to that of males (26, 27).

In an *in vitro* model, we stimulated whole blood with lipopolysaccharide (LPS) and pokeweed mitogen (PWM) from prepubertal children of both sexes and Turner syndrome patients, the latter expressing a female phenotype but carrying an X0 genotype, thus more similar to men (28). Cytokine secretion (IL-1β and IL-6) was higher in the males than females, and Turner syndrome patients followed the same pattern of response as the males, suggesting the X chromosome potentially plays a role.

Clinically, women express higher inflammation than men of any age (7, 18, 19, 29–31), while most *in vitro* studies report higher inflammatory cytokine production in men than women (28, 32–35). These discrepancies could result from sex-specific differences in the kinetics or sensitivity of immune receptors, such as TLRs, and demonstrate the multiple interactions taking place between the immune system and other organs.

To evaluate the influence of sex chromosomes, we previously studied several immune functions linked to the X chromosome in healthy adults (33). In whole blood stimulated with LPS, we observed a higher secretion of tumor necrosis factor (TNF)-α and a tendency to produce more IL-6 in men compared to women. Stimulating purified neutrophils, however, revealed no inter-sex difference in terms of cytokine production, indicating that neutrophils probably do not play a central role in the primary response (33).

In order to evaluate the respective contribution of the X chromosome and sex hormones, we measured herein the activation of some TLRs according to X-linked genes in subjects with different X/Y sex chromosome ratios, including subjects with Klinefelter's syndrome XXY. The purpose of our study was to verify if males carrying an extra X chromosome were susceptible to present a similar cytokine secretion pattern compared to women despite their different levels of sex steroids and to identify eventually the proteins of the TLR signaling pathway responsible for the sex differences that we observed previously.

Patients with Klinefelter syndrome are phenotypically males but carry an extra X chromosome. Their karyotype includes therefore 47 chromosomes with three sex chromosomes XXY. Klinefelter syndrome is the most frequent aneuploidy in males with a prevalence estimated to about 150 per 100,000 males. Patients present with tall stature, small testes, gynecomastia, language impairment, infertility and hypergonadotropic hypogonadism (36). Since most of these features rarely present in one individual, the disease is often missed, especially during the first years of life (37). Their expected life span is reduced by 1 to 2 years, mainly due to metabolic syndrome, lung disease, epilepsy, cerebrovascular disease and breast cancer (38). Several studies reported an increased risk of autoimmune diseases in patients with Klinefelter's syndrome such as Addison's disease, multiple sclerosis, diabetes mellitus, thyroiditis, rheumatoid arthritis, Sjogren's syndrome or lupus erythematosus, similar to women (39–42).

We focused on certain TLR signaling pathways and what resulted from their activation using specific ligands, as TLRs are evolutionarily conserved and crucial for the innate immune response. TLRs recognize a broad range of lipids, carbohydrates, peptides, and nucleic acids expressed by microorganisms. Mutations and polymorphisms of genes involved in TLR signaling pathways are responsible for congenital immunodeficiency disorders and susceptibility to infectious diseases.

Finally, we sought to identify which leukocyte population plays a predominant role in the kinetics of the sex-specific response to early inflammation.

## Materials and Methods

### Reagents

Highly-purified lipopolysaccharide (LPS) from *Escherichia coli* 026:B6 was purchased from Sigma-Aldrich (St. Louis, Missouri) to stimulate TLR4, while Zymosan (TLR2 ligand) and Resiquimod (TLR7/8 ligand) where purchased from InvivoGen (San Diego, California). Polymorphprep™ was purchased from Axis-Shield (Oslo, Norway) to separate polymorphonuclear neutrophils (PMNs) from peripheral blood mononuclear cells (PBMCs). All monoclonal antibodies used for flow cytometry were acquired from Becton-Dickinson (BD) Biosciences (San Jose, California). To avoid estrogens in culture media, we used Hank's balanced salt solution (HBSS) containing Ca^2+^ and Mg^2+^, in addition to phenol-red free Roswell Park Memorial Institute medium (RPMI) 1640 containing L-glutamine medium and, charcoal stripped and dextran treated, fetal bovine serum (FBS) from Invitrogen Life Technologies (Paisley, UK).

Any possibility of endotoxin presence from external sources in reagents and assay material was excluded using the chromogenic LAL Endotoxin Assay Kit from GenScript (Piscataway, New Jersey).

The doses used for stimulation were those recommended by the manufacturer and tested to obtain a sub-maximal response on the dose-response curve.

### Subjects

Blood samples were taken from healthy Caucasian adult patients with Klinefelter syndrome and healthy Caucasian adult donors of both sexes. As estrogen levels display a wide distribution in women depending on their age and menstrual cycle stage, only those taking combined hormonal oral contraceptives were selected so as to standardize the group's estrogens levels (43). Subjects with Turner syndrome were excluded after the initial analysis due to the high proportion of mosaic patients.

For each experiment, 17 subjects of each group were analyzed; one person of each group being sampled the same day. Analyses were performed in duplicate from the same sample.

The study was approved by the *Hôpital Erasme* ethics committee (reference: P2010/221), and all human participants provided written informed consent.

### Blood Samples

All blood samples were obtained by means of venipuncture of the forearm vein, each collected in 10 tubes containing 3.2% of sodium citrate and two containing 68 units of sodium heparin. The tubes were purchased from Becton-Dickinson (BD) Diagnostics (Franklin Lakes, New Jersey), and each batch was tested for the presence of endotoxin.

### Preparation of Human Neutrophils

Neutrophils were isolated from citrated blood samples by means of gradient centrifugation using Polymorphprep™. After washing, cell pellets with over 98% neutrophils on May-Grünwald Giemsa-stained cytopreparations were resuspended in RPMI 1640 medium containing L-glutamine, penicillin, streptomycin, and 10% fetal bovine serum (FBS). A cell count was performed using Advia 2120 (Siemens Healthcare Hematology System, Omaha, Nebraska).

### Preparation of Human Monocytes

After separating the PBMCs from citrated blood samples by means of gradient centrifugation, we isolated the CD14+ cells using the CD14 microbeads from Miltenyi Biotec (Bergisch Gladbach, Germany). Briefly, the CD14 cells were labeled with anti-CD14 microbeads then loaded onto a MACS™ column placed in the magnetic field of a MACS™ separator, retaining the magnetically-labeled CD14+ cells. After washing, the cell pellets exhibiting over 95% monocytes on May-Grünwald Giemsa-stained cytopreparations, were resuspended in RPMI 1640 medium containing L-glutamine, penicillin, streptomycin, and 10% fetal bovine serum (FBS). A complete cell count was performed using Advia 2120 (Siemens Healthcare Hematology System, Omaha, Nebraska).

### Inflammatory Cytokine Production

Whole blood samples were mixed in a 1:5 ratio with RPMI 1640 medium containing L-glutamine, penicillin, streptomycin, and 10% FBS, then incubated, respectively, with LPS (TLR4 agonist) or Resiquimod (TLR7/8 agonist) at a final concentration of 1 μg/mL, and Zymosan (TLR2 agonist) at a final concentration of 10 μg/mL for 24 h in a humidified incubator at 37°C with 5% CO_2_ (Heraeus HBB 2472b, Heraeus Instrument GmbH, Hanau, Germany). In the control samples, TLR ligand volumes were replaced by RPMI.

Purified PMNs and purified monocytes were resuspended at 2 × 10^6^ cells/mL and 0.2 × 10^6^ cells/mL, respectively, in the same culture medium and incubated in the same conditions as the whole blood.

The supernatant was collected after centrifugation (800 g for 5 min) and frozen at −80°C until assay. Production of IL-1β, IL-6, IL-8, IL-10, TNF-α, and interferon (IFN)-α were measured by flow cytometry using the Cytometric Bead Array Human Cytokine Kit from BD Biosciences (San Jose, California).

### Phosphorylation of TLR Signaling Pathway Proteins in Whole Blood

Following stimulation with LPS, the intracellular quantity of the phosphorylated forms of NF-κB p65, ERK1/2, and p38 MAPK was evaluated in monocytes and PMNs using flow cytometry. Whole blood was stimulated with LPS at 10 μg/mL for 15 min at 37°C, corresponding to the time to reach the peak of phosphorylation. Reactions were stopped, and red blood cells removed at the same time using BD Phosflow Lyse/Fix Buffer (BD Biosciences, San Jose, California). After washing, cells were permeabilized using BD Phosflow Perm Buffer (BD Biosciences, San Jose, California) and incubated on ice for 30 min. The cells were mixed with phycoerythrin (PE)-conjugated monoclonal antibodies (mAb) against phosphorylated nuclear factor-kappaB (NF-κB) p65 (pS529), Alexa Fluor 488 (A488)-conjugated mAb against phosphorylated ERK1/2 (pT202/pY204), phycoerythrin (PE)-cyanin 7 (Cy7)-conjugated mAb against phosphorylated p38 MAPK (pT180/pY182), and allophycocyanin (APC)-conjugated mAb against CD33 for 1 h at room temperature. The samples were assayed on a FACS Canto II flow cytometer (BD Biosciences, San Jose, California). Leukocyte populations were differentiated based on side scatter (SSC) and CD33 expression. We measured the median fluorescence intensity of the fluorochrome for each leukocyte population before and after stimulation.

### Phosphorylation of TLR Signaling Pathway Proteins in Purified Monocytes

The concentration of phosphorylated proteins of the TLR signaling pathway was measured in purified monocytes following stimulation of the cell suspension by TLR ligands and lysis. Purified monocytes, resuspended at 10 × 10^6^ cells/mL in RPMI 1640 medium containing L-glutamine and 10% FBS, were incubated with either LPS (TLR4 agonist) or Zymosan (TLR2 agonist), respectively, at a final concentration of 10 μg/mL, or Resiquimod (TLR7/8 agonist) at a final concentration of 1 μg/mL, for 15 min in a humidified incubator at 37°C with 5% CO_2_ (Heraeus HBB 2472b, Heraeus Instrument GmbH, Hanau, Germany). In the control samples, TLR ligand volume was replaced by RPMI. After 15 min, the reaction was stopped by adding ice-cold PBS and cells were pelleted. The cell pellet was resuspended with denaturation buffer, and the samples placed in a boiling water bath for 5 min to denature the proteins. Cell lysates were frozen at −80°C until assay. We measured concentrations of the following phosphorylated kinases: extracellular signal-regulated kinase (ERK) 1/2 (T202/Y204), p38 mitogen-activated protein kinase (MAPK) (T180/Y182), and c-Jun N-terminal kinase (JNK) 1/2 (pT183/pY185), using the Cytometric Bead Array Human Cell Signaling Kit from BD Biosciences (San Jose, California).

### Estradiol and Testosterone

Total 17β-estradiol (E2) and total testosterone (T) were measured in serum by direct radioimmunoassay (RIA), obtained from Diasorin (Saluggia, Italy).

### Statistical Analysis

The data were analyzed by the principal investigator, Nicolas Lefèvre, and all authors had access to primary data.

Continuous variables were expressed as means and standard error of the mean (SEM). Student's *t*-test for independent groups was used to compare men and women. To analyze the influence of the sex chromosome balance, one-way ANOVA was performed on each observed variable with one group factor (karyotype) at three levels (XX, XXY, XY), followed by Tukey's tests or T3 Dunnett tests, if needed, in cases of variance heterogeneity so as to compare the three karyotypes.

Results were expressed as mean ± SEM, and statistical significance was set at *p* < 0.05. We used IBM-SPSS statistical software for Windows, Version 23.0 (IBM Corp., Armonk, NY). The ^*^, ^**^, and ^***^ symbols, respectively, signify *p* < 0.05, *p* < 0.01, and *p* < 0.001.

#### Statistical Adjustments

To test the possible impact of differences in sex steroid levels, the data were evaluated a second time following normalization of steroid levels. Therefore, one–way ANCOVA tests were performed on each observed variable with one group factor (karyotype) at three levels (XX, XXY, XY), and with estradiol and testosterone as covariates, in order to compare the three karyotypes while adjusting for the sex steroid levels.

## Results

We compared 17 healthy Caucasian subjects with Klinefelter syndrome (XXY). Patients with Klinefelter syndrome were older (mean age: 39 ± 8 years) than women (XX) (mean age: 28 ± 4 years) and men (XY) (mean age: 30 ± 5 years) (*p* < 0.001). No comorbidities were noted in any of the groups.

### Inflammatory Cytokine Production in Whole Blood (Table 2)

#### Response to LPS

IFN-α secretion was not detected after stimulation with LPS.

**Figure 1:**
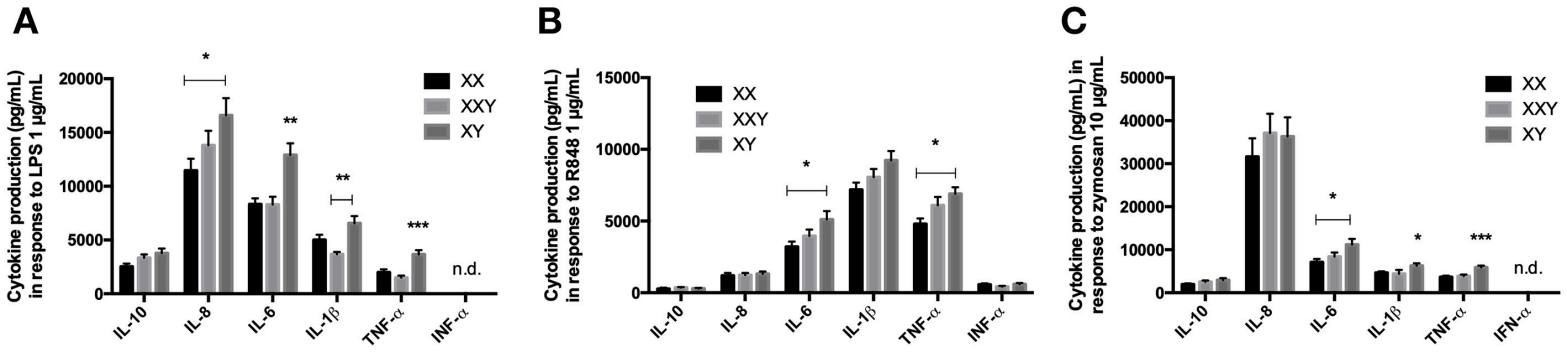
Mean ± SEM of IL-10, IL-8, IL-6, IL-1β, TNF-α, and IFN-α productions in whole blood after stimulation with LPS 1 μg/mL **(A)**, resiquimod 1 μg/mL **(B)** and zymosan 10 μg/mL **(C)** in men, women, and subjects with Klinefelter syndrome. Whole blood was mixed with culture medium then incubated with the ligand for 24 h. The production of inflammatory cytokines was measured in the supernatant by flow cytometry. BD FACSDiva™ software version 6.1.2 for Windows was used to acquire the data and BD FCAP Array™ software version 1.0.1 for Windows was used to analyze the data. (*p < 0.05, **p < 0.01, ***p < 0.001 using one-way ANOVA for independent groups with *n* = 17).

When compared to men, women exhibited significantly lower production of IL-10 (*p* = 0.020), IL-8 (*p* = 0.012), IL-6 (*p* < 0.001), and TNF-α (*p* = 0.002). IL-1β tended to be lower in women too, yet the difference was not statistically significant (*p* = 0.066) (Figure 1A).

When we analyzed the three groups together, IL-8 production was significantly lower in women than men (16,605 ± 1,598 pg/mL in men vs. 11,461 ± 1,106 pg/mL in women, *p* = 0.028), IL-6 production was significantly lower in both women and subjects with Klinefelter (12,914 ± 1,078 pg/mL in men vs. 8,320 ± 555 pg/mL in women, *p* = 0.003; 8,290 ± 727 pg/mL in subjects with Klinefelter, *p* = 0.004), IL-1β production was significantly lower in subjects with Klinefelter (6,565 ± 662 pg/mL in men vs. 3,679 ± 220 pg/mL in subjects with Klinefelter, *p* < 0.001), and TNF-α production was significantly lower in both women and subjects with Klinefelter (3,651 ± 1,707 pg/mL in men vs. 1,990 ± 279 pg/mL in women, *p* = 0.001; 1,494 ± 787 pg/mL in subjects with Klinefelter, *p* < 0.001) (Figure 1A).

#### Response to Resiquimod

We observed significantly lower production of IL-6 (*p* = 0.009), IL-1β (*p* = 0.017), and TNF-α (*p* = 0.002) in women compared to men (Figure 1B).

When evaluating all three karyotypes, production of IL-6 (5,109 ± 580 pg/mL in men vs. 3,207 ± 365 pg/mL in women, *p* = 0.023) and TNF-α (6,891 ± 469 pg/mL in men vs. 4,794 ± 393 pg/mL in women, *p* = 0.013) was significantly lower in women compared to the men but IFN-α levels were not significantly different between the three groups (Figure 1B).

#### Response to Zymosan

No IFN-α secretion was detected after stimulation with zymosan.

Women produced significantly less IL-6 (*p* < 0.001), IL-1β (*p* = 0.025), and TNF-α (*p* < 0.001) than men when stimulated with zymosan (Figure 1C).

When we compared the three groups together, cytokine production was lower in women compared to men for IL-6 (11,200 ± 1,305 pg/mL in men vs. 7,122 ± 713 pg/mL in women, *p* = 0.018) and lower in both women and subjects with Klinefelter compared to men for IL-1β (6,263 ± 587 pg/mL in men vs. 4,630 ± 366 pg/mL in women, *p* = 0.043; 4,377 ± 415 pg/mL in subjects with Klinefelter, *p* = 0.017) and TNF-α (5,901 ± 427 pg/mL in men vs. 3,665 ± 318 pg/mL in women, *p* < 0.001; 3,856 ± 382 pg/mL in subjects with Klinefelter, *p* = 0.001) (Figure 1C).

### Inflammatory Cytokine Production by Purified Monocytes (Table 3)

#### Response to LPS

**Figure 2:**
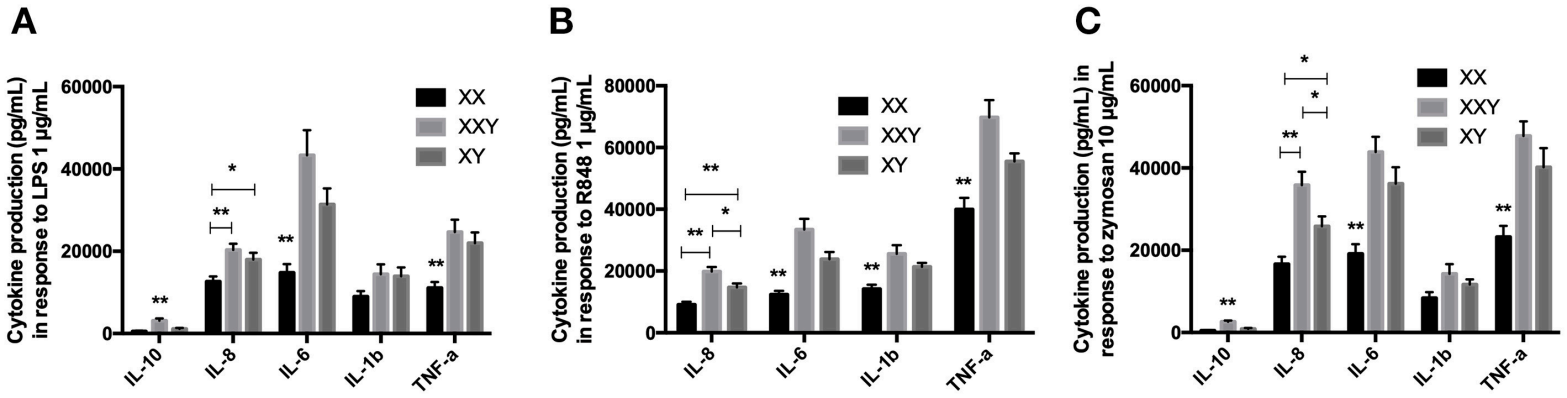
Mean ± SEM of IL-10, IL-8, IL-6, IL-1β, and TNF-α productions by purified monocytes after stimulation with LPS 1 μg/mL **(A)**, resiquimod 1 μg/mL **(B)** and zymosan 10 μg/mL **(C)** in men, women, and subjects with Klinefelter syndrome. Purified monocytes resuspended at 0.2 × 10^6^ cells/mL in culture medium were incubated with the ligand for 24 h. The production of inflammatory cytokines was measured in the supernatant by flow cytometry. BD FACSDiva™ software version 6.1.2 for Windows was used to acquire the data and BD FCAP Array™ software version 1.0.1 for Windows was used to analyze the data. (*p < 0.05 and **p < 0.01 using one-way ANOVA for independent groups with *n* = 17).

Compared to men, women exhibited significantly lower production of IL-10 (*p* = 0.026), IL-8 (*p* = 0.015), IL-6 (*p* < 0.001), and TNF-α (*p* < 0.001). Production of IL-1β also tended to be lower in women in comparison with men, though the difference did not reach statistical significance (*p* = 0.062) (Figure 2A).

When we analyzed the three groups together, IL-10 production was significantly lower in women and men compared to subjects with Klinefelter (501±154 pg/mL in women, *p* < 0.001, and 1,141 ± 226 pg/mL in men, *p* = 0.012 vs. 3,110 ± 571 pg/mL in subjects with Klinefelter), IL-8 production was significantly lower in women compared to both men and subjects with Klinefelter (126,480 ± 12,464 pg/mL in women vs. 179,658 ± 16,398 pg/mL in men, *p* = 0.036; 203,161 ± 14,917 pg/mL in subjects with Klinefelter, *p* = 0.002), as was IL-6 production (14,753 ± 2,143 pg/mL in women vs. 31,368 ± 3,889 pg/mL in men, *p* = 0.003; 43,311 ± 6,132 pg/mL in subjects with Klinefelter, *p* = 0.001) and TNF-α production (11,097 ± 1,449 pg/mL in women vs. 21,987 ± 2,583 pg/mL in men, *p* = 0.003; 24,658 ± 3,016 pg/mL in subjects with Klinefelter, *p* = 0.001) (Figure 2A).

#### Response to Resiquimod

IL-10 production by purified monocytes in response to resiquimod was under the detection limit of our kit.

We observed significantly lower production of IL-8 (*p* = 0.001), IL-6 (*p* < 0.001), IL-1β (*p* < 0.001), and TNF-α (*p* = 0.002) in women compared to men (Figure 2B).

When evaluating all three karyotypes, we observed lower IL-8 production in women and men compared to subjects with Klinefelter, as well as in women compared to men (91,276 ± 9,380 pg/mL in women, *p* < 0.001; 146,689 ± 12,901 pg/mL in men, *p* = 0.013 vs. 198,439 ± 14,340 pg/mL in subjects with Klinefelter). IL-6 (12,358 ± 1,218 pg/mL in women vs. 23,818 ± 2,369 pg/mL in men, *p* = 0.001; 33,462 ± 3,411 pg/mL in subjects with Klinefelter, *p* < 0.001), IL-1β (14,144 ± 1,448 pg/mL in women vs. 21,337 ± 1,306 pg/mL in men, *p* = 0.003; 25,565 ± 2,809 pg/mL in subjects with Klinefelter, *p* = 0.004), and TNF-α production (39,963 ± 3,770 pg/mL in women vs. 55,502 ± 2,611 pg/mL in men, *p* = 0.006; 69,794 ± 5,565 pg/mL in subjects with Klinefelter, *p* < 0.001) was significantly lower in women in comparison with men and subjects with Klinefelter (Figure 2B).

#### Response to Zymosan

Women produced significantly less IL-8 (*p* = 0.004), IL-6 (*p* < 0.001), and TNF-α (*p* = 0.003) than men when stimulated with zymosan (Figure 2C).

When we compared the three groups together, cytokine production was lower in women and men compared to subjects with Klinefelter for IL-10 (491 ± 111 pg/mL in women, *p* < 0.001 and 898 ± 206 pg/mL in men, *p* < 0.001 vs. 2,602 ± 325 pg/mL in subjects with Klinefelter). IL-8 secretion was lower in both women and men compared to subjects with Klinefelter and especially lower in women compared to men (16,603 ± 18,419 pg/mL in women, *p* < 0.001 and 258,344 ± 23,752 pg/mL in men, *p* = 0.022 vs. 358,223 ± 32,679 pg/mL in subjects with Klinefelter, *p* < 0.001). Productions of IL-6 (19,142 ± 2,346 pg/mL in women vs. 36,187 ± 4,011 pg/mL in men, *p* = 0.003; 43,868 ± 3,688 pg/mL in subjects with Klinefelter, *p* < 0.001) and TNF-α (23,221 ± 2,682 pg/mL in women vs. 40,174 ± 4,644 pg/mL in men, *p* = 0.012; 47,751 ± 3,650 pg/mL in subjects with Klinefelter, *p* < 0.001) was significantly lower in women compared to either men or subjects with Klinefelter (Figure 2C).

### Inflammatory Cytokine Production by Purified Neutrophils

**Figure 3:**
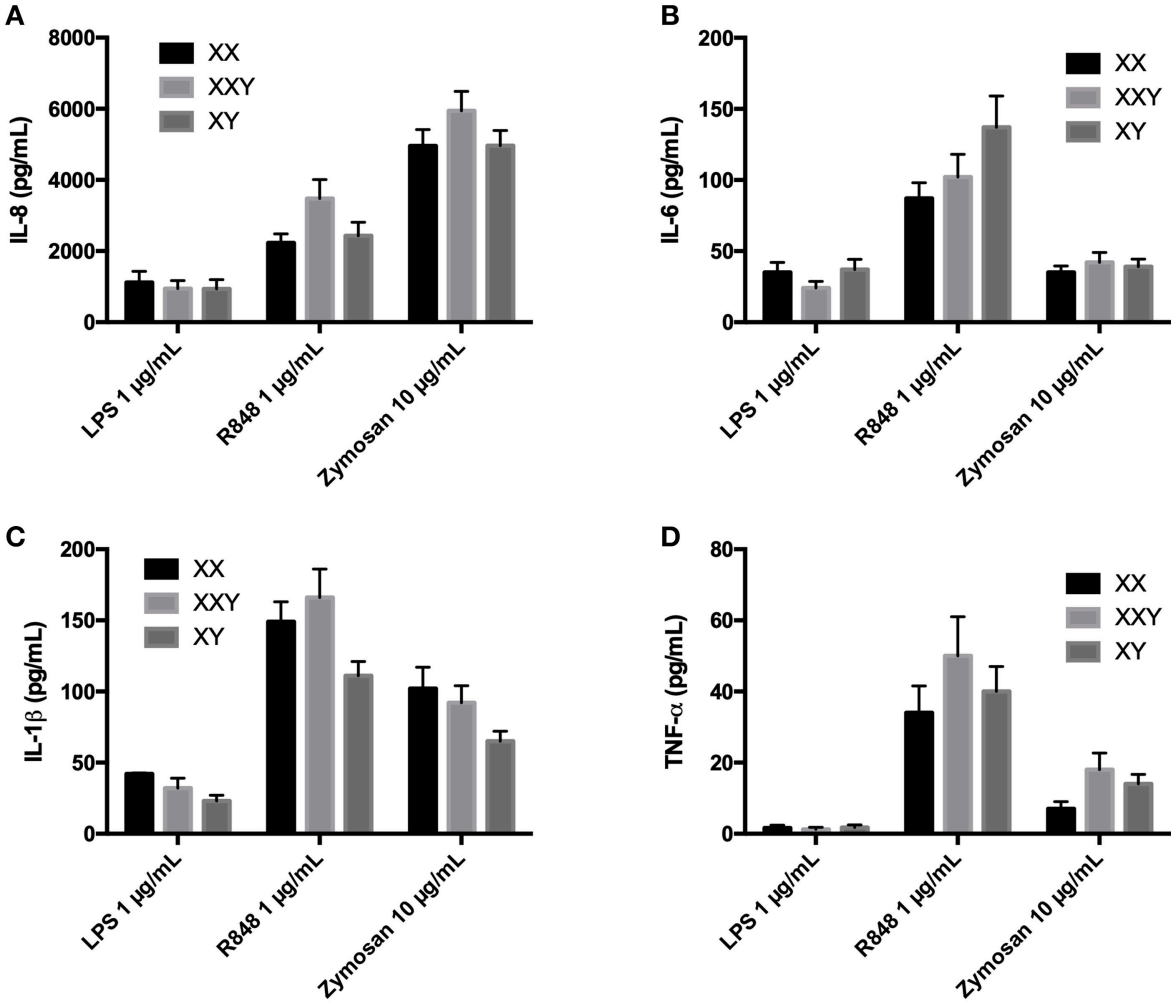
Mean ± SEM of IL-8 **(A)**, IL-6 **(B)**, IL-1β **(C)**, and TNF-α **(D)** productions in purified neutrophils after stimulation with LPS 1 μg/mL, resiquimod 1 μg/mL and zymosan 10 μg/mL in men, women, and subjects with Klinefelter syndrome. Purified neutrophils resuspended at 2 × 10^6^ cells/mL in culture medium were incubated with the ligand for 24 h. The production of inflammatory cytokines was measured in the supernatant by flow cytometry. BD FACSDiva™ software version 6.1.2 for Windows was used to acquire the data and BD FCAP Array™ software version 1.0.1 for Windows was used to analyze the data.

No significant difference between women, men, and subjects with Klinefelter were identified (Figure 3).

### Phosphorylation of TLR Signaling Pathway Proteins in Purified Monocytes

**Figure 4:**
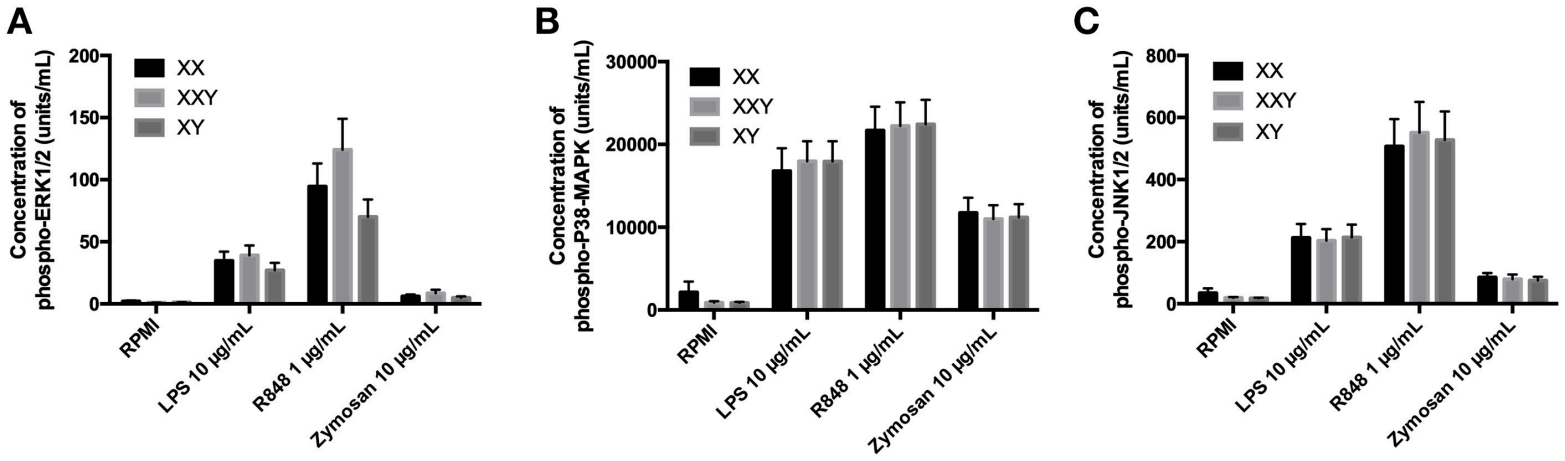
Mean ± SEM of phospho-ERK1/2 **(A)**, phospho-P38-MAPK **(B)**, and phospho-JNK1/2 **(C)** concentrations in purified monocytes after stimulation with LPS 10 μg/mL, resiquimod 1 μg/mL and zymosan 10 μg/mL in men, women, and subjects with Klinefelter syndrome. Purified monocytes resuspended at 10 × 10^6^ cells/mL in culture medium were incubated with the ligand for 15 min. Concentrations of the phosphorylated kinases were measured in the cell lysate by flow cytometry. BD FACSDiva™ software version 6.1.2 for Windows was used to acquire the data and BD FCAP Array™ software version 1.0.1 for Windows was used to analyze the data.

We observed no difference between groups in terms of concentration of the proteins p-ERK (Figure 4A), p-38 (Figure 4B), and p-JNK (Figure 4C) in response to any of the three TLR ligands tested.

### Phosphorylation of TLR Signaling Pathway Proteins in Whole Blood

**Figure 5:**
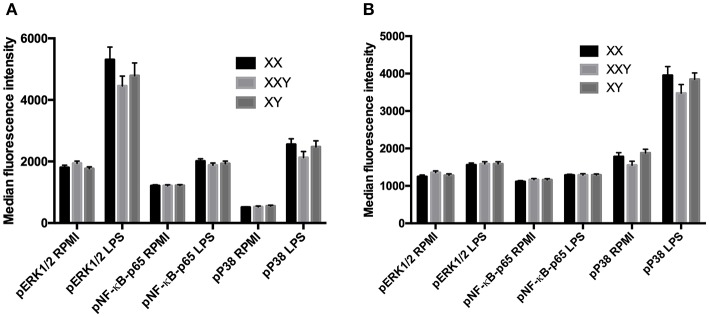
Mean ± SEM of the median fluorescence intensity of phospho-ERK1/2, phospho-NF-κB-p65 and phospho-P38-MAPK in monocytes **(A)** and neutrophils **(B)**, before and after stimulation of whole blood with LPS 10 μg/mL in men, women, and subjects with Klinefelter syndrome. Whole blood was stimulated with LPS at 10 μg/mL for 15 min. Reactions were stopped, and red blood cells removed. After washing, cells were permeabilized and mixed with fluorochrome-conjugated monoclonal antibodies against the phosphorylated kinases. The median fluorescence intensity of each fluorochrome was measured in each leukocyte population by flow cytometry. BD FACSDiva™ software version 6.1.2 for Windows was used to acquire the data and BD FCAP Array™ software version 1.0.1 for Windows was used to analyze the data.

Following stimulation with LPS, the levels of phosphorylation of ERK, p38, and NF-κB did not significantly differ between men, women, and subjects with Klinefelter, either in monocytes (Figure 5A) or neutrophils (Figure 5B).

### Estradiol and Testosterone

**Figure 6:**
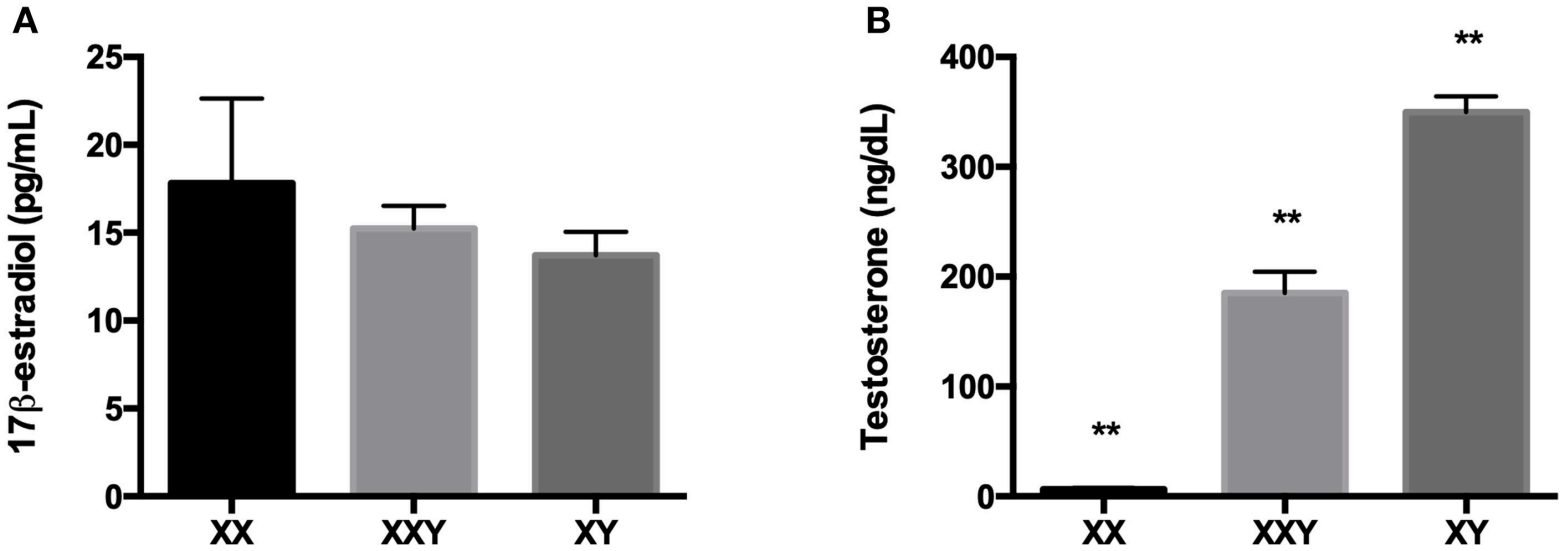
Mean±SEM of the levels of 17β-estradiol **(A)** and testosterone **(B)** in the serum of women (XX), subjects with Klinefelter syndrome (XXY), and men (XY). Sex steroids were measured in serum by direct radioimmunoassay (RIA). (**p < 0.01 using one-way ANOVA for independent groups with *n* = 17).

There was no significant difference in 17β-estradiol levels between groups (*p* = 0.62). Testosterone levels were significantly different across all groups (*p* < 0.01), with Klinefelter subjects exhibiting higher levels than women (185 ± 19.2 ng/mL for Klinefelter subjects vs. 6.5 ± 1.4 ng/mL for women, *p* < 0.01) yet lower than men (349.8 ± 14.4 ng/mL, *p* < 0.01) (Figure 6).

### General Blood Parameters

As already described for other cohorts, the women in our study exhibited significantly lower hemoglobin levels compared to men and Klinefelter subjects (*p* = 0.003 compared to men and *p* = 0.015 compared to Klinefelter subjects), in addition to significantly lower monocyte counts (*p* = 0.033 compared to men and *p* = 0.021 compared to Klinefelter subjects). Women also had significantly less red blood cells (*p* = 0.010) and less eosinophils (*p* = 0.036) compared to men. There was no significant difference between groups for other blood parameters (Table 1).

**Table 1:** Mean ± SEM for complete blood count (*n* = 17).

**Blood parameters (unit)**	**XX**	**XXY**	**XY**	**p**
Red blood cells (× 10^6^/μL)	4.62 ± 0.14	4.92 ± 0.09	5.06 ± 0.06	0.011^*^
Hemoglobin (g/dL)	13.7 ± 0.4	14.9 ± 0.2	15.1 ± 0.2	0.002^**^
Platelets (× 10^3^/μL)	169 ± 13	153 ± 7	145 ± 12	0.321
White blood cells (× 10^3^/μL)	5.91 ± 0.32	5.88 ± 0.40	5.80 ± 0.21	0.969
Neutrophils (× 10^3^/μL)	3.33 ± 0.23	3.29 ± 0.35	3.38 ± 0.18	0.972
Eosinophils (× 10^3^/μL)	0.12 ± 0.01	0.20 ± 0.03	0.18 ± 0.02	0.031^*^
Basophils (× 10^3^/μL)	0.06 ± 0.01	0.12 ± 0.07	0.04 ± 0.01	0.365
Lymphocytes (× 10^3^/μL)	2.00 ± 0.16	1.80 ± 0.15	1.70 ± 0.08	0.285
Monocytes (× 10^3^/μL)	0.27 ± 0.01	0.38 ± 0.03	0.36 ± 0.02	0.007^**^

* p < 0.05 and

** *p < 0.01 using one-way ANOVA for independent groups with n = 17*.

### Inflammatory Cytokine Production in Whole Blood After Statistical Adjustment for Estradiol and Testosterone Levels (Table 2)

Following correction for the potential effects of estradiol and testosterone, all differences between groups disappeared, except for IL-1β (*p* = 0.004) and TNF-α (*p* = 0.029) production in response to LPS, being significantly lower in subjects with Klinefelter in comparison with women.

**Table 2:** Comparison of p values obtained with one-way ANOVA with one group factor (karyotype) at three levels (XX, XXY, XY), and p values obtained with one–way ANCOVA with estradiol and testosterone as covariates, performed on the levels of cytokines secreted in whole blood after stimulation with LPS, resiquimod and zymosan.

		**ANOVA with one factor (karyotype)**			**ANCOVA with two covariates (steroids)**		
		**XX vs. XY**	**XX vs. XXY**	**XY vs. XXY**	**XX vs. XY**	**XX vs. XXY**	**XY vs. XXY**
LPS	IL-10	ANOVA not significant (*p* = 0.055)			0.276	0.465	0.262
	IL-8	0.028	0.454	0.325	0.18	0.295	0.219
	IL-6	0.003	1.000	0.004	0.754	0.125	0.277
	IL-1β	0.072	0.138	<0.001	0.831	0.004	0.076
	TNF-α	0.001	0.496	<0.001	0.707	0.029	0.093
	IFN-α	Not detectable			Not detectable		
R848	IL-10	ANOVA not significant (*p* = 0.626)			0.713	0.85	0.404
	IL-8	ANOVA not significant (*p* = 0.876)			0.218	0.326	0.269
	IL-6	0.018	0.494	0.206	0.569	0.479	0.81
	IL-1β	ANOVA not significant (*p* = 0.052)			0.976	0.898	0.952
	TNF-α	0.01	0.151	0.487	0.664	0.582	0.863
	IFN-α	ANOVA not significant (*p* = 0.292)			0.367	0.117	0.917
Zymosan	IL-10	ANOVA not significant (*p* = 0.123)			0.314	0.421	0.367
	IL-8	ANOVA not significant (*p* = 0.641)			0.405	0.319	0.695
	IL-6	0.018	0.643	0.138	0.807	0.576	0.868
	IL-1β	0.043	0.922	0.017	0.338	0.069	0.778
	TNF-α	<0.001	0.932	0.001	0.624	0.126	0.435
	IFN-α	Not detectable			Not detectable		

### Inflammatory Cytokine Production by Purified Monocytes After Statistical Adjustment for Estradiol and Testosterone Levels (Table 3)

#### Response to LPS

When we adjusted our results for estradiol and testosterone levels, differences remained for IL-6 (*p* = 0.024 for women vs. men; *p* < 0.001 for women vs. subjects with Klinefelter) and TNF-α (*p* = 0.011 for women vs. men and *p* = 0.001 for women vs. subjects with Klinefelter) production, and IL-10 production was still lower in women compared to subjects with Klinefelter (*p* = 0.001), while IL-1β production also became significantly lower in women compared to subjects with Klinefelter (*p* = 0.033).

**Table 3:** Comparison of *p*-values obtained with one-way ANOVA with one group factor (karyotype) at three levels (XX, XXY, XY), and *p*-values obtained with one–way ANCOVA with estradiol and testosterone as covariates, performed on the levels of cytokines secreted by purified monocytes after stimulation with LPS, resiquimod, and zymosan.

		**ANOVA with one factor (karyotype)**			**ANCOVA with two covariates (steroids)**		
		**XX vs. XY**	**XX vs. XXY**	**XY vs. XXY**	**XX vs. XY**	**XX vs. XXY**	**XY vs. XXY**
LPS	IL-10	0.076	<0.001	0.012	0.231	0.001	0.084
	IL-8	0.036	0.002	0.499	0.939	0.147	0.162
	IL-6	0.003	0.001	0.292	0.024	<0.001	0.903
	IL-1β	ANOVA not significant (*p* = 0.123)			0.07	0.033	0.372
	TNF-α	0.003	0.001	0.875	0.011	0.001	0.469
Resiquimod	IL-10	Not detectable			Not detectable		
	IL-8	0.007	<0.001	0.013	0.024	<0.001	0.375
	IL-6	0.001	<0.001	0.079	0.253	0.001	0.095
	IL-1β	0.003	0.004	0.45	0.025	0.001	0.862
	TNF-α	0.006	<0.001	0.084	0.18	0.002	0.246
Zymosan	IL-10	0.253	<0.001	<0.001	0.844	0.001	0.001
	IL-8	0.037	<0.001	0.022	0.014	<0.001	0.674
	IL-6	0.003	<0.001	0.262	0.006	<0.001	0.774
	IL-1β	ANOVA not significant (*p* = 0.064)			0.368	0.08	0.758
	TNF-α	0.012	<0.001	0.49	0.003	<0.001	0.506

#### Response to Resiquimod

Following correction for the potential effects of estradiol and testosterone, we still observed lower production of all cytokines in women compared to subjects with Klinefelter (*p* < 0.001 for IL-8, *p* = 0.001 for IL-6, *p* = 0.001 for IL-1β, and *p* = 0.002 for TNF-α), as well as lower IL-8 (*p* = 0.024) and IL-1β (*p* = 0.025) production in women compared to men.

#### Response to Zymosan

After correcting for the potential effects of estradiol and testosterone, differences persisted for all cytokines, except for IL-8 production which differed between men and Klinefelter patients.

IL-10 production was lower in women and men compared to subjects with Klinefelter (*p* = 0.001), and IL-8 production was lower in women compared to men (*p* = 0.014) and subjects with Klinefelter (*p* < 0.001). Production of IL-6 (*p* = 0.006 for women vs. men and *p* < 0.001 for women vs. subjects with Klinefelter) and TNF-α (*p* = 0.003 for women vs. men and *p* < 0.001 for women vs. subjects with Klinefelter) remained lower in women compared to men and subjects with Klinefelter.

## Discussion

Our study highlighted significant differences between the innate immune responses of men and women by measuring the products of TLR signaling pathway activation. Some of these differences cannot be explained by estradiol and testosterone levels.

As already reported in previous *in vitro* studies, especially in response to LPS (28, 33–35), we observed higher levels of inflammatory cytokines in men compared to women after stimulating whole blood with various TLR ligands, reflecting the activation of the innate immune system by fungal (TLR2), bacterial (TLR4), and viral (TLR7/8) products. When compared with subjects with Klinefelter syndrome who are phenotypically male but carry an extra X chromosome like women, inflammatory responses in whole blood were similar to that observed in females, probably as a consequence of the X chromosome mosaicism on the X-linked genes involved in the TLR signaling pathway.

Although Klinefelter patients exhibited slightly lower testosterone levels compared to men, yet much higher than women, their cytokine profile appeared similar to that of women, suggesting that sex chromosomes are more influential than sex steroids, as previously observed with Turner syndrome patients. Indeed, subjects with Turner syndrome, who are phenotypically female but carry only one X chromosome, presented a mirror image of the same pattern of cytokine production as males (28).

Males might suffer of a process of inbreeding depression on the proteins coded by the X chromosome, including the kinases of the TLR signaling pathway, due to their single X chromosome in comparison with females and subjects with Klinefelter syndrome who are carrying both two X chromosomes, and thus susceptible to select the most advantageous polymorphisms for these genes (22, 24). The lower secretion of inflammatory cytokines in women may protect them from an uncontrolled and potentially life-threatening immune response in cases of severe aggression such as sepsis, trauma or extensive burns throughout life (44, 45).

When analyzing each phagocyte population separately, we found no difference in neutrophils between the sexes. In purified monocytes, activation of TLRs produced lower levels of inflammatory cytokines in women compared to men, as observed in whole blood. Yet in contrast with whole blood analyses, the purified monocytes of Klinefelter subjects expressed the same pattern of cytokine production as that found in males. These results observed in purified monocytes, and previously reported with peripheral blood mononuclear cells in response to TLR7 (46), would suggest that monocytes are playing an essential role in the sex-specific innate immune response but are probably not the cells responsible for the differences observed in whole blood. Indeed, TLR stimulation in whole blood leads to activation of many other cells including from the adaptive immune system as well as platelets and proteins potentially able to modulate the cytokine secretion through activation of other X-linked genes such as the CD40 ligand or the CD99. The inflammatory cytokine secretion pattern of Klinefelter subjects resembled that of women in whole blood, thus confirming our hypothesis, yet differed in purified monocytes.

We observed no difference in levels of phosphorylated mitogen-activated protein kinase (MAPK) or phosphorylated transcription factor NF-κB expression, which are involved in different TLR4 signaling pathways, as previously described in neutrophils (35). Although the sensitivity of our method could have been too low to detect small variations in phosphorylation levels of these proteins, the differences in cytokine production could also result from downstream mechanisms in the transcription phase.

In whole blood, after correcting statistically for the potential effects of estradiol and testosterone, women expressed higher levels of IL-1β and TNF-α in response to TLR4 stimulation than subjects with Klinefelter. In monocytes, most of the differences remained even after correcting for estradiol and testosterone levels, indicating there to be no priming or persistent effect from the sex steroids, given that the culture medium of the purified monocytes was free of hormones. These results support claims that the X chromosome contributes to sex-related differences in cytokine secretion, independently of levels of the two main sex hormones.

Likewise, unpublished data from our group revealed that increasing concentrations of 17β-estradiol had no significant effect on inflammatory cytokine production in whole blood of prepubertal children after stimulation with LPS. Although the sex-specific response of the TLRs seems to be based on gene expression, it is probably regulated by the hormonal environment, hence the discrepancies after adjusting for estradiol and testosterone levels (47). In this context, it is important to remember that hormonal levels are determined by karyotype. Therefore, statistical correction of estradiol and testosterone as covariates could have artificially modified the results, assuming both hormone levels and immune parameters are equally modulated by sex chromosome balance. Considering these parameters, the whole blood results with no statistical correction are, in fact, the closest to what could be observed *in vivo*.

As already observed in our previous study (33) and described in a larger study including 200 Caucasian subjects (48), we also noticed a significantly higher monocyte count in patients carrying a Y chromosome. However, these differences, as well as the higher eosinophil count observed in Klinefelter subjects, disappeared after adjusting for sex steroid levels. In another study comparing the complete blood cell count of 86 healthy prepubertal children (43 girls and 43 boys aged 2 to 6 years), we observed no significant difference in leucocyte populations between the sexes (results not published). These results indicate the possibility that this difference in monocyte count might be related to sex hormones and is not the main cause of the sex-specific inflammatory responses observed throughout life.

Our study revealed higher inflammatory response in men compared to women and subjects with Klinefelter syndrome, both of whom carry two copies of the X chromosome and thus potentially benefit from cellular mosaicism in X-linked genes.

Sex-specific secretion of these cytokines in response to the activation of TLR4 may be thus influenced by polymorphisms in X-linked genes and therefore cellular mosaicism in females. This mechanism could give them an advantage in acute inflammatory processes, though is likely to lead to poorer prognosis in chronic inflammatory diseases, resulting in increased inflammatory damages even early in life.

## Ethics Statement

The study was approved by the *Hôpital Erasme* ethics committee (reference: P2010/221), and all human participants provided written informed consent.

## Author Contributions

NL drafted the manuscript. NL, FC, JV, and AD carried out the experiments. NL, FC, AD, and GC designed the study and participated in its coordination. JD participated in the design of the study. VD performed statistical analysis. All authors read and approved the final manuscript.

### Conflict of Interest Statement

The authors declare that the research was conducted in the absence of any commercial or financial relationships that could be construed as a potential conflict of interest.
